# Outer Membrane Proteins as Vaccine Targets Against *Lawsonia intracellularis* in Piglets

**DOI:** 10.3390/vaccines13020207

**Published:** 2025-02-19

**Authors:** Kara L. Aves, Ana H. Fresno, Sajid Nisar, Mauro M. Saraiva, Nicole B. Goecke, Adam F. Sander, Morten A. Nielsen, John E. Olsen, Priscila R. Guerra

**Affiliations:** 1 Department of Immunology and Microbiology, Centre for Medical Parasitology, University of Copenhagen, Blegdamsvej 3B, 2200 Copenhagen, Denmarkasander@sund.ku.dk (A.F.S.); mortenn@sund.ku.dk (M.A.N.); 2 Department of Veterinary and Animal Sciences, University of Copenhagen, Stigbøjlen 4, 1800 Frederiksberg, Denmark; sanisar@sund.ku.dk (S.N.); mauro.saraiva@unesp.br (M.M.S.); nbgo@sund.ku.dk (N.B.G.); jeo@sund.ku.dk (J.E.O.); 3 Departamento de Bioquímica y Biología Molecular, Facultad de Ciencias, Campus Terra, Universidad de Santiago de Compostela (USC), 27002 Lugo, Spain; 4 Department of Pathology, Reproduction, and One Health, Universidade Estadual Paulista-Unesp-FCAV, Prof. Paulo Donato Castellane s/n, Jaboticabal 14884-900, Brazil

**Keywords:** pigs, antigen, virus-like particle, enteropathy, diarrhea, OMP2, MBP.INVASc

## Abstract

Background: *Lawsonia intracellularis* (LI) is the agent of proliferative enteropathy in swine, a common disease that affects pigs for up to eight weeks after weaning. Aim: To evaluate the effectiveness of two novel subunit vaccines targeting outer membrane proteins on LI. Methods: The two vaccines included OMP2c.cVLP, where the OMP2c antigen was anchored on the surface of capsid virus-like particles (cVLP); and MBP.INVASc, where antigens were anchored to an MBP fusion protein. Groups of six mice, as proof of concept, and six piglets were immunized with either OMP2c.cVLP, MBP.INVASc., or PBS as a control using a prime-boost regime. Results: Both OMP2c.cVLP and MBP.INVASc subunit vaccines induced strong antigen-specific serum IgG and IgA responses. There were no significant differences in weight gain among the groups. Mild-to-moderate clinical signs of LI infection were observed, but vaccinated groups showed lower inflammatory scores and fewer animals tested positive for bacteria by immunohistochemistry. Although neither vaccine completely prevented clinical signs of LI infection, both effectively reduced inflammation and lowered the pathogen load, thereby mitigating the severity of the disease, particularly the MBP.INVASc vaccine. Conclusions: These findings suggest that both vaccines have the potential for further development and optimization to enhance their protective efficacy against LI infections.

## 1. Introduction

Enteric pathogens pose a significant threat to livestock, compromising animal health and welfare and undermining production efficiency. Traditional treatments, reliant on antibiotics, face increasing challenges due to the emergence of antimicrobial resistance, prompting the necessity for the development of novel preventive or therapeutic approaches, including vaccines [1]. *Lawsonia intracellularis* (LI) causes proliferative enteropathy, resulting in the thickening of the intestinal epithelial cells of pigs, and these changes are highly correlated with the intracellular presence of the pathogen [2]. LI is endemic worldwide, resulting in significant economic losses for swine producers due to decreased growth rates, increased mortality, and the additional expenses associated with antimicrobial treatment [3,4]. Infection often leads to diarrhea, progressing from watery to bloody, and stunted weight gain, which can be fatal, especially in young pigs [4,5].

Currently, commercial live-attenuated and inactivated bacterin-based vaccines are available for LI prophylaxis. The live-attenuated vaccine, commercially available since 2001, requires strict management practices such as an antibiotic-free feed window [6]. The immunity produced by such a vaccine seems efficient but is still less robust than the immunity conferred by natural infection [4,7,8]. Even though some benefits, such as improved weight gain and reduced antimicrobial use, have been demonstrated, the live attenuated commercially available vaccine still does not fully prevent clinical disease and transmission [9]. The inactivated vaccine, on the other hand, has no restrictive practices but tends to generate a lower immune response [8], which remains suboptimal compared to natural infection. Considering that both vaccines do not provide full protection and have drawbacks [6,8], further research into new vaccine approaches is necessary. Recent immunoproteomic studies have identified outer membrane proteins, such as OMP2c and INVASc, which are targets of naturally acquired immunity [10]. These proteins have shown protective efficacy when used as subunit vaccines, although previous studies have used co-formulation of the antigens. Recent research for other pathogens has developed vaccines based on capsid virus-like particles (cVLPs), combined with a unique technique to anchor antigens on the surface of the capsid [11]. The repetitive surface geometry of cVLPs facilitates strong B cell receptor crosslinking and subsequent B cell activation. The structural mimic of the native virions enables efficient lymphatic drainage, uptake by antigen-presenting cells, and activation of the innate immune system [11,12]. Therefore, cVLPs are potentially valuable as scaffolds for antigen display in vaccine development. In these constructs, vaccine antigens are expressed and purified as genetic fusions with a short split-protein binding tag, facilitating their attachment onto pre-assembled cVLPs. Upon mixing with cVLPs presenting the corresponding binding partner (a Catcher protein), a covalent isopeptide bond is formed between the Tag and antigen. A traditional approach is to link the antigen to a maltose-binding protein (MBP) fusion. This enhances the stability and solubility of the target antigen, which is particularly useful for antigens with poor solubility [13]. The current study aimed to compare the humoral immune response of OMP2c and INVASc in their native structural context and when applied as subunit vaccines in mice, as a proof of concept, and piglets to assess their protective efficacy against LI.

## 2. Materials and Methods

### 2.1. Protein Expression and Purification

OMP2c (amino acids 24-241, excluding N-terminal secretion signal and the OmpA-like transmembrane C-terminal domain to facilitate soluble protein expression) from the PHE/MN1-00 LI genome (GenBank: WP_011526985.1) was engineered with an N-terminal SpyCatcher (amino acids 25-139 of GenBank: AFD50637.1 [14]. OMP2c (amino acids 24-241, excluding the OmpA-like C-terminal domain) from the PHE/MN1-00 LI genome (GenBank: WP_011526985.1) was engineered with an N-terminal SpyCatcher (amino acids 25-139 of GenBank: AFD50637.1) and a 6xHis-tag, separated by a Gly-Gly-Ser-Gly flexible linker. NcoI and NotI restriction sites were added to the N- and C-terminus, respectively, to facilitate cloning into the pET-15b vector. INVASc (amino acids 22-244) from the PHE/MN1-00 genome (GenBank: WP_011526924) was designed with a C-terminal Gly-Gly-Ser-Gly flexible linker followed by the SpyTag sequence (AHIVMVDAYKPTK). NcoI and NotI restriction sites were used for cloning into the pETM-41 vector, placing the gene immediately downstream of a 6xHis-tag and the maltose-binding protein (MBP) gene.

#### 2.1.1. Purification of Tag.cVLP

*Acinetobacter* phage AP205 coat protein subunit (Gene ID: 956335), displaying an N-terminal SpyTag (Tag.cVLP; Gene ID: OK545878.1), was expressed and purified as previously described [15]. Briefly, expression was performed in *E. coli* One Shot^®^ BL21 (DE3) cells (Invitrogen, Waltham, MA, USA), and particles were purified by ultracentrifugation using an Optiprep™ (Sigma-Aldrich, St. Louis, MI, USA) density step gradient. Optiprep was removed by a single O/N dialysis at 4 °C against 5L 1xPBS using a 1000 kDa MWCO dialysis tube (Spectrum Labs, Los Angeles, CA, USA), and protein concentration was determined by BCA assay according to the manufacturer’s instructions (Thermo Scientific, Waltham, MA, USA).

#### 2.1.2. Formulation and Quality Assurance of cVLP Vaccine

Before formulation, Catcher.OMP2c and cVLPs were LPS purified using Triton X-114 and the phase separation technique. OMP2c.cVLP was formulated by mixing Tag.cVLP with Catcher.OMP2c at a 1:1.75 molar ratio and incubated O/N at 4 °C. Excess unbound antigen was removed by ultracentrifugation using an Optiprep density step gradient followed by a single O/N dialysis at 4 °C against 5L 1xPBS. Coupling efficiency and size distribution were assessed as stated [11]. Samples were diluted to 0.2–0.4 mg/mL, centrifuged at 16,000× *g* for 2 min, and loaded into an Eppendorf Uvette cuvette (Sigma-Aldrich, St. Louis, USA). Measurements were performed using a DynaPro NanoStar (WYATT Technology, Santa Barbara, CA, USA) with refractive index set to the PBS standard, with temperature set at 25 °C collecting 20 acquisitions of 5 s each. Triplicate samples were analyzed. Average hydrodynamic diameter, percentage polydispersity (%Pd), and particle integrity were evaluated. Samples (0.1–0.3 mg/mL) were adsorbed onto glow-discharged grids for 1 min, which were then washed with ultra-pure water, stained with 2% uranyl acetate for 1 min, and imaged using a CM 100 BioTWIN microscope (Philips, Amsterdam, The Netherlands) [11]. MBP.INVASc failed to form stable (non-aggregated) particles when coupled to the cVLP; thus, the soluble antigen was used for immunizations.

### 2.2. Challenge Dose Preparation

Inoculum material was prepared from a clinically affected pig with LI, confirmed by real-time PCR (qPCR) (see section qPCR analysis). To exclude the presence of other intestinal pathogens, additional diagnostic evaluations were conducted targeting common enteric pathogens such as, e.g., *Salmonella* spp., Enterotoxigenic *E.coli*, *Brachyspira* spp. These tests confirmed the absence of co-infection. The mucosa from the small intestine walls of this animal was scraped and washed two times with PBS, following a modified protocol based on Boesen et al. 2004 [16]. Mucosa tissue (10% homogenized) was either used directly as inoculum or the suspension was transferred into gelatin 200 µL capsules (size 1) (CapsulCN- Ruian, China). The capsules were snap-frozen using dry ice and stored at −20 °C until the time of inoculation. Each animal received one capsule containing approximately 10^7^ CFU daily for three consecutive days.

### 2.3. Murine Immunization Studies

Female C57BL/6j AnNRj mice (six to eight weeks old) were obtained from Janvier Labs (Le Genest-Saint-Isle, France), and housed in a specific pathogen-free facility. Mice (n = 6 per group) were immunized intranasally with OMP2c.cVLP at an antigen dose of 7 µg or MBP.INVASc at an antigen dose of 20 μg. Three immunizations were given at two-week intervals. Serum samples were collected 13 days after each vaccine. Subsequently, all mice were dosed with 1 mL of homogenized (10%) mucosa from a pig infected with LI, administered for three consecutive days. Dulbecco’s modified eagle medium (DMEM) (negative control) and non-vaccinated LI (positive control) were included. The behavior and health status of the animals were monitored over the trial. At the end of the experiment, mice were sacrificed by cervical dislocation and necropsied. Three mice per group were randomly selected for histopathological analysis.

### 2.4. Immunization Study of Piglets

Eighteen (18) piglets, approximately two weeks old, were obtained from non-immunized Danish crossbreed sows and underwent CLP immunization. These piglets were purchased from a pig production herd in Denmark, with selection based on a birthweight of 1–2 kg and a lack of prior disease history in the sows. Both male and female piglets were randomly included in the selection process. The selected piglets were divided into three groups, identified with earmarks (six pigs per group). Before vaccination, serum samples were taken from six randomly selected piglets. Piglets were then administered the initial immunization of either OMP2c.cVLP + AddaVax™ (Copenhagen, Denmark) adjuvant or MBP.INVASc + AddaVax™ adjuvant. All vaccines were given at a dose of 100 µg of total protein. A Control group was included, in which animals received a mock vaccination with PBS saline solution. One piglet from the control group died before the challenge. On the weaning day (17 days post prime), piglets were transported to the experimental facility at the University of Copenhagen, Frederiksberg, Denmark. Upon arrival, each piglet received a booster shot of the immunization according to their immunization plan. Serum samples were collected 24 days post-prime immunization (seven days post-boost).

The piglets were housed in separate rooms according to their assigned groups, with each pen featuring solid concrete floors and sawdust bedding. The facility maintained negative ventilation pressure and included a clean room for waste management. Daily waste, including used bedding, was collected in plastic bags and disinfected to prevent contamination. Strict hygiene protocols were followed, including complete changes of clothes, hairnets, gloves, and boots upon entry and exit from the rooms. Boots were disinfected each time, and handwashing and surface cleaning were conducted regularly. No materials were shared between rooms to prevent cross-contamination. Ventilation was maintained at a rate of ten air exchanges per hour, while temperature control kept the environment within 20–24 °C. Additionally, piglets had access to a heated resting area maintained at 30–32 °C. Throughout the study, piglets were provided ad libitum access to standard commercial feed without antibiotics, zinc, organic acids, or blood plasma.

Twelve (12) days after the booster, each animal was administered a capsule containing the LI challenge, with dosing repeated for three consecutive days. Piglets were weighed on the challenge day and on the termination day (12 days post-challenge). Post-challenge clinical examinations and fecal consistency scoring were conducted on days five, eight, and twelve. Fecal scoring followed a scale, in which 0 represented normal feces, 1 indicated mild diarrhea, 2 signified moderate diarrhea, and 3 indicated severe diarrhea or the presence of blood, following the criteria by Jacobs et al. [17]. The body condition scoring was 0 for normal, while 1 indicated visible gaunt or sunken sides, and 2 was assigned when animals exhibited protruding spine and rib bones. Gross pathology assessments were conducted after termination, involving inspection of stomach contents, inspection of the small and large intestine, and evaluation of the ileocecal valve. Fragments from the distal ileum and mid-colon were collected for histopathological examination, while fecal content from the distal ileum, colon, and cecum was sampled for qPCR analysis.

### 2.5. qPCR Analysis

LI was detected in the fecal swab and intestinal content samples via qPCR following the protocol described by Ståhl et al. [18]. Briefly, the purified DNA using the QIAcube HT extraction robot and the Cador Pathogen 96 QIAcube HT kit (QIAGEN, Hilden, Germany) was analyzed on the Rotor-Gene Q qPCR system (QIAGEN, Hilden, Germany) under the thermal cycling conditions: 94 °C for 2 min, followed by 40 cycles of 94 °C for 15 s and 60 °C for 60 s. Data were analyzed with Rotor-Gene Q series software, version 2.3.5 (QIAGEN). Each extraction and PCR run included positive and negative controls (nuclease-free water). An analytical cut-off value of cycle threshold (Ct) 36 was used for the data analysis.

A positive plasmid standard containing a specific gene fragment of LI was designed and purchased from Eurofins Genomics (Ebersberg, Germany). A ten-fold dilution series of the positive standard was made in PBS, and subsequently, each dilution was spiked in 10% LI-negative feces, and DNA was extracted as described above. The extracted DNA from each dilution (10% negative feces) was analyzed in triplicates and separate qPCR runs, and a standard curve ranging from 7.85 × 10^10^ to 7.85 × 10^2^ copies no./g feces was constituted based on these results. The linear range and efficiency of the qPCR assay were determined. In each qPCR analysis of the samples, a dilution of the standard curve was included as a positive control, facilitating adjustment of the standard curve to each new qPCR run.

### 2.6. Serology

Serology was assessed as described previously [11]. Briefly, an ELISA was used to measure antigen-specific antibody responses in mouse and pig serum. The 96-well plates (Nunc MaxiSorp, Thermo Fisher, Waltham, MA, USA) were coated with 0.1 µg/well of recombinant MBP.INVASc and MBP.OMP2c(aa24-241), followed by O/N incubation at 4 °C. Plates were blocked with 0.5% skimmed milk in PBS for 1 h at RT. Serum samples were diluted in PBS + 0.5% skimmed milk, using a three-fold dilution starting at 1:50 for IgG or a two-fold dilution starting at 1:20 for IgA. To determine seropositivity, pig samples were run in parallel with a non-immune control serum (31890, Invitrogen, Carlsbad, CA, USA). Fecal extracts were prepared to measure total IgA in PBS + 1% BSA. Sample dilutions were inoculated into the plates, further incubated for 1 h at room temperature (RT), washed, probed with HRP-conjugated secondary antibodies (anti-mouse IgG, anti-pig IgG, and anti-pig IgA), washed an additional three times, and developed with TMB X-tra substrate (Kem-En-Tec, Copenhagen, Denmark). Absorbance was measured at 450 nm, and antibody levels were reported as the area under the curve (AUC) [11].

### 2.7. Histopathology

Intestinal tissue samples obtained from mice and pigs were fixed in 10% neutral buffered formalin phosphate (Fisher Scientific, Roskilde, Denmark) and subsequently processed for histological examination. After routine processing and paraffin embedding, tissue sections were sliced at 4 µm thickness and stained with hematoxylin and eosin (H&E). Microscopic analysis was conducted using an Olympus BX45 light microscope equipped with a DP25 digital camera (Olympus, Søborg, Denmark). For the immunohistochemistry (IHC), sections (circa 3 μm thick) of tissue samples were cut onto SuperFrost Plus slides (Menzel-Glaser, Braunschweig, Germany), deparaffinized in xylene, rehydrated through graded alcohols, and air-dried. Endogenous peroxidase was blocked by treating sections with 3% hydrogen peroxide solution for 20 min followed by two washes in Tris-buffered saline (TBS), pH 7.5–7.6. All slides were then subjected to Ultra V block (Thermo Fisher, Waltham, MA, USA) for 5 min. Then, the primary antibody, Law1-DK (BIO 323 Bio-X Diagnostics), diluted at 1:1000, and antibody number 2, IgG1 (DAKO X0931), diluted at 1:219 in TBS, were added, followed by two TBS washes. Then, Ultravision ONE HRP polymer (Thermo Fisher, Waltham, MA, USA) was applied for 30 min, followed by two washes with TBS. All sections were incubated in 3,3′-diaminobenzidine (DAB) substrate solution (Sigma Aldrich, St. Louis, MI, USA) for 10 min and then rinsed twice with distilled water. Slides were then stained with hematoxylin and mounted with glycerol-gelatine. Sections were evaluated for inflammation according to the following criteria: (0) no significant lesions; (1) up to 25% hyperplastic enterocytes, focal or multifocally, with a reduction in the number of goblet cells; (2) 25 to 50% hyperplastic enterocytes, multifocally, with reduced goblet cell numbers; (3) 50 to 75% hyperplastic enterocytes, multifocally, with decreased goblet cell numbers; and (4) more than 75% hyperplastic enterocytes, multifocally or diffusely, with reduced goblet cell numbers. All sections were blinded to treatment, and histopathological assessments were conducted by a certified veterinary pathologist affiliated with the University of Copenhagen. IHC evaluation adhered to the protocol outlined by Boesen et al. [17].

### 2.8. Haptoglobin

The serum concentration of haptoglobin (Hp) was measured using an ELISA kit (ab223136, Abcam, Cambridge, UK) specifically designed for the quantitative analysis of haptoglobin in porcine serum. All samples were tested in duplicate and processed blindly. Based on the literature, the baseline range for healthy piglets was established between 0.6 and 1.4 mg/mL [19,20].

### 2.9. Statistical Analysis

Statistical analyses were performed using GraphPad Prism version 8.4.3 (GraphPad, San Diego, CA, USA). An unadjusted, non-parametric, two-tailed Mann–Whitney U test was applied to compare the differences between the two groups. For comparisons involving multiple groups, we utilized a one-way ANOVA with a Dunnett correction to account for multiple comparisons. For all statistical tests, a *p*-value < 0.05 was considered statistically significant.

### 2.10. Ethical Statement

All animal experiments conducted in this study were ethically approved by the Danish Animal Experiments Inspectorate (Dyreforsøgstilsynet) under approval numbers 2020-15-0201-00465 and 2018-15-0201-01541. The experiments were carried out in strict adherence to national Danish guidelines and the high standards of animal welfare established by FELASA (Federation of European Laboratory Animal Science Associations).

## 3. Results

### 3.1. Vaccine Quality Control

**Figure 1:**
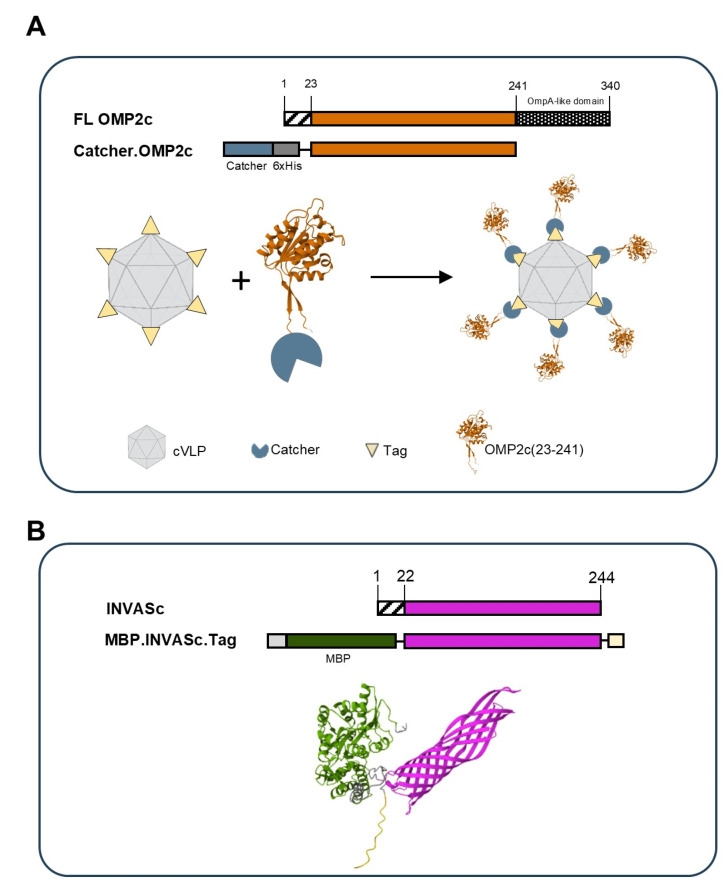
Vaccine design: (**A**) Schematic representation of OMP2c antigen design. Full-length (FL) OMP2c consists of an N-terminal secretion signal and C-terminal OmpA-like domain. OMP2c aa 24-241 was N-terminally fused to a Catcher sequence and 6xHis tag to form Catcher.OMP2c. Recombinant Catcher.OMP2c was mixed with Tag.cVLPs, and the resulting covalent conjugation between the Tag and Catcher led to the unidirectional display of the antigen in the OMP2c.cVLP vaccine. (**B**) Schematic representation of INVASc antigen design. Full-length INVASc, excluding the secretion signal, was expressed as a genetic fusion to MBP. Protein structural predictions were generated by Alphafold version v2.3.0 and visualized using Mol*.

To enhance solubility and facilitate stable coupling of OMP2c to cVLPs, the N-terminal domain of the protein (i.e., excluding the OmpA-like domain) was expressed in *E. coli* as a genetic fusion to the Catcher protein (Figure 1A). OMP2c.Catcher was conjugated to cVLPs via the Tag/Catcher interaction, and the final vaccine was monodispersed (%Pd of 13.4), displaying high levels of particle stability (Supplementary Figure S1). INVASc is a chimeric β-barrel structure protein [8] and therefore difficult to express as a soluble protein in *E. coli.* After failed attempts to solubilize and refold the protein from inclusion bodies, we found that fusion to MBP carrier protein generated the most stable protein, with the greatest proportion of protein present in the soluble cell fraction. Although a split-protein binding tag was added to the C-terminal of the protein, MBP.INVASc did not bind effectively or stably to the cVLP particles. Consequently, we used the soluble MBP.INVASc protein alone in subsequent immunization studies.

### 3.2. Immunogenicity and LI Challenge in a Murine Model

**Figure 2:**
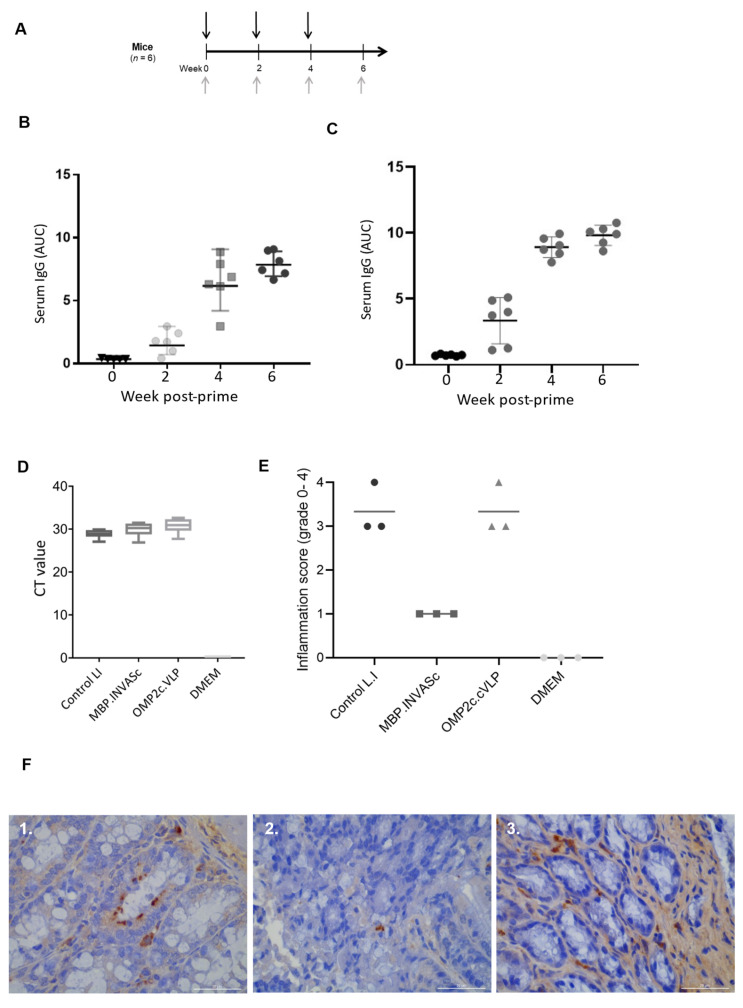
Induction of humoral immune responses in mice: (**A**) Experimental setup. Mice (n = 6) were immunized intranasally with either OMP2c.cVLP or MBP.INVASc (black arrows). Serum was collected before immunization (day 0) and each boost (grey arrows). ELISA measured anti-OMP2c (**B**) and anti-MBP-INVASc (**C**) specific serum IgG. Each point represents one animal, and the bars show the mean area under the curve (AUC) antibody titer ± SD at each time point. (**D**) Median inflammation score post-*Lawsonia intracellularis* challenge. (**E**) qPCR results (Ct level) of *L. intracellular* detection in colon samples. (**F**) 1. Intestinal fragments of a control mouse showing anti-LI staining in the epithelium of the intestinal crypts. 2. Intestinal fragment of a vaccinated mouse anti-(MBP-INVASc) showing anti-LI staining in the epithelium of the intestinal crypts (streptavidin–biotin), ×40 magnification; arrows indicate immunopositive red-stained *L. intracellularis*. 3. Intestinal fragment of a vaccinated mouse anti-(OMP2c-VLP) showing anti-LI staining in the epithelium of the intestinal crypts (streptavidin–biotin), ×40 magnification; arrows indicate immunopositive red-stained *L. intracellularis*.

The murine model challenge was conducted to determine if mice could mount an immune response after immunization with OMP2c.cVLP and MBP.INVASc subunit vaccines, followed by a challenge with LI. We observed that both OMP2c.cVLP and MBP.INVASc subunit vaccines induced strong antigen-specific serum IgG responses (Figure 2). Mice vaccinated with MBP.INVASc subunit vaccinate showed less inflammation (grade 1) when compared to the LI group upon challenge with infective LI (Figure 2D). In contrast, all mice from the OMP2c group had higher inflammation scores of 3 or 4, similar to those in the LI group (Figure 2D). This indicates that the OMP2c.cVLP subunit vaccine did not reduce the inflammation levels in mice. Additionally, both vaccines (MBP.INVASc and OMP2c.cVLP) did not prevent pathogen shedding (Figure 2D). The results were further supported by IHC staining (Figure 2E).

### 3.3. Immunogenicity and Protective Efficacy in Piglets

**Figure 3:**
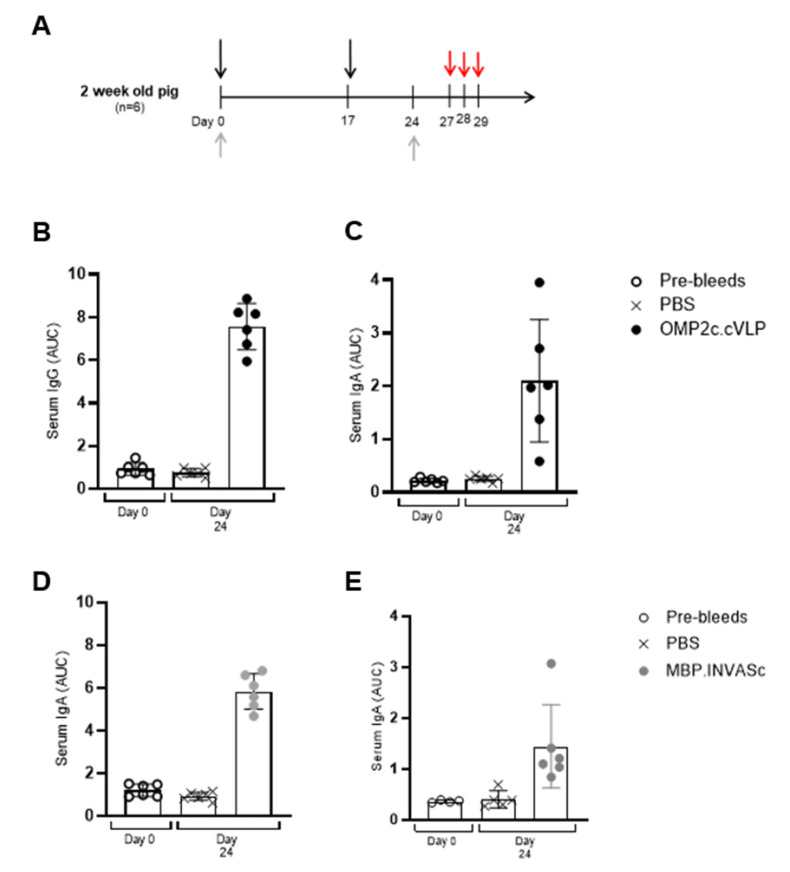
Induction of humoral immune response after intramuscular vaccination in piglets. (**A**) Experimental setup. Suckling piglets (n = 6) were immunized twice intramuscularly with either PBS vehicle, OMP2c.cVLP, or MBP.INVASc (black arrows). Serum was collected before immunization (day 0) and seven days after the boost (day 24) (grey arrows), and the pig received three days of challenge starting from 27 days post prime immunization. Anti-OMP2c (**B**,**C**), anti-MBP-INVASc (**D**,**E**), specific serum IgG (**B**,**D**), and serum IgA (**C**,**E**) responses in the piglets were measured by ELISA. Each point represents one animal, and the bars show the mean area under the curve (AUC) antibody titer ± SD at each time point.

After the mice model assay, we transitioned to a target species, aiming to obtain more relevant insights into vaccine efficacy and immune response in the intended population. In this experiment, we evaluated the immune response of pigs immunized with the subunit vaccines, OMP2c.cVLP or MBP.INVASc, and subsequent challenge with LI. Suckling piglets were given an intramuscular (i.m) prime-boost immunization using these vaccines (OMP2c.cVLP or MBP.INVASc), formulated with an adjuvant. The immunized pigs showed a robust induction of antigen-specific serum IgG and IgA antibodies compared to the PBS-vaccinated control pigs, demonstrating a strong immune response (Figure 3B–E), thus highlighting the enhanced antibody production in the vaccinated groups.

### 3.4. Weight Gain, Body Conditions, and Inflammation Markers in the Pig Experiment

**Figure 4:**
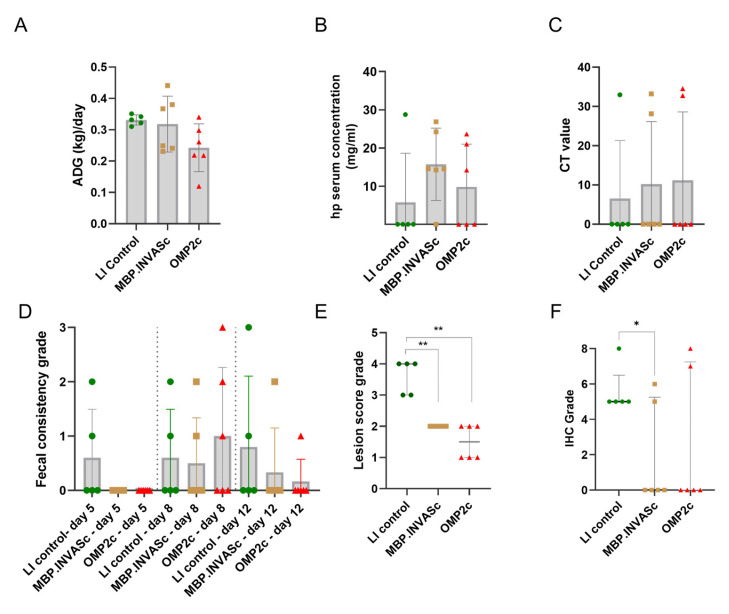
Weight gain, body conditions, fecal consistency, and inflammation markers observed in pigs: (**A**) average daily gain (ADG); (**B**) serum haptoglobin concentration levels on the termination day; (**C**) qPCR results Ct level of LI in fecal swabs on the termination day (0 = a negative result in the qPCR analysis); (**D**) fecal consistency grade on days 5, 8, and 12; (**E**) median value of microscopic lesion in the ileum post-LI challenge; (**F**) median IHC staining scores of ileum fragments; * *p*-value < 0.05.

In terms of relative weight gain, no significant differences were observed among the pigs in the control and vaccination groups (Figure 4A). The control group averaged a gain of 368 g daily, the MBP.INVASc group gained 353 g daily, while the OMP2c group had a slightly lower average daily gain of 270 g (Figure 4A). Regarding the post-challenge body condition, one piglet from the LI control group and one from the OMP2c group showed a body condition score of 1, indicating mild dehydration. Despite these cases, no signs of severe dehydration were detected in any of the animals. After the challenge, most animals exhibited mild-to-moderate clinical signs of LI, such as soft feces or watery diarrhea, following the criteria described by Jacobs et al. [17]. In the vaccinated groups, three out of six animals in the OMP2c group displayed symptoms, including one case of moderate-to-severe diarrhea on day eight post-challenge. In the control group, two out of five animals showed symptoms such as mild signs of dehydration and diarrhea (Figure 4D). Other instances of diarrhea were classified as mild to moderate. Overall, our observations demonstrated that the vaccines induced robust humoral responses but did not prevent the appearance of clinical signs of LI infection.

Haptoglobin is a protein that binds to free hemoglobin released from red blood cells, thereby preventing oxidative activity and protecting tissues from damage [20]. The MBP.INVASc-vaccinated group exhibited a slightly elevated haptoglobin level at their endpoint, although these differences were not statistically significant (Figure 4B).

**Figure 5:**
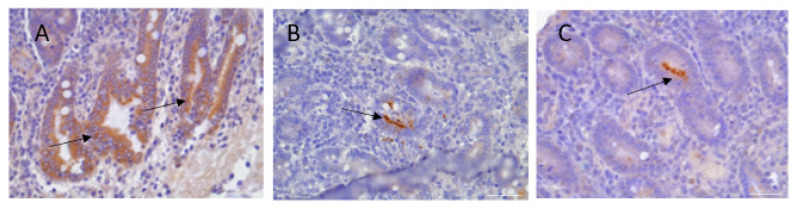
Pig intestinal fragment showing anti-LI staining in the epithelium of the intestinal crypts. Streptavidin–biotin, ×40 magnification; arrows indicate immunopositively red-stained LI. (**A**) Animal from the control group; (**B**) animal from the MBP.INVASc-vaccinated group; (**C**) animal from the OMP2c.cVLP-vaccinated group.

A gross pathology examination was performed on the termination day, and subsequently, histopathology analysis was performed to determine the presence of inflammation. No macroscopic lesions were observed in the small or large intestines of either the vaccinated or control groups of LI. However, microscopic lesions were observed in all animals from the control group, which scored either grade 3 or 4, and those observations represented significantly increased pathology in comparison with the animals from the vaccinated groups, which scored grades ranging from 1 to 2 (Figure 4E). LI antigen was detected by IHC staining in 100% of the animals from the LI control group, with grading scores ranging from five to eight (Figure 4F and Figure 5). In contrast, in the LI-vaccinated groups, MBP.INVASc and OMP2c, the antigen was detected in only two out of six animals in both groups, with scores ranging from five to eight (Figure 4F). Low detection levels in fecal swab samples were observed among both vaccinated groups (Figure 4C). Ct values correspond to 3.6 × 10^4^ and 1.43 × 10^6^ copies no./g feces (MBP.INVASc); 1.39 × 10^4^ and 4.93 × 10^4^ copies no./g feces (OMP2c.cVLP), and 4.28 × 10^4^ copies no./g feces (LI control groups).

## 4. Discussion

*Lawsonia intracellularis* remains one of the leading pathogens responsible for enteric disease in pigs and continues to have a high global prevalence [21]. As regulations on the usage of antimicrobials in the swine industry are becoming more controlled, the demand for effective prophylactic options is increasing. Therefore, this study aimed to evaluate the immunogenicity and protective efficacy of two potential vaccine candidates, OMP2c.cVLP and MBP.INVASc, against *LI* infection in both murine and porcine models.

Our findings provide valuable insights into the potential use of the subunit vaccines, OMP2c.cVLP and MBP.INVASc, to mitigate inflammatory responses and bacterial load. In our in vivo studies, both subunit vaccines demonstrated immunogenicity. Presenting the OMP2 antigen on the cVLP surface is expected to increase its immunogenicity due to better recognition and processing by the immune system. Displaying poorly immunogenic vaccine antigens on the surface of cVLP is a well-validated method to increase antigen immunogenicity, resulting in enhanced antibody responses [11]. Similarly, a recent study using cVLP targeting F4 enterotoxigenic *E. coli* demonstrated that cVLP might potentially improve the humoral response against low-immunogenic antigens in piglets, particularly if these are not targeted by pre-existing maternal immunity [11].

MBP.INVASc did not show effective or stable binding cVLP particles, leading us to use the unconjugated MBP.INVASc protein in subsequent immunization studies. Despite this challenge, we were able to generate a stable and soluble protein fraction when fused to MBP. The MBP-fusion was chosen because it can enhance the solubility and proper folding of target proteins, and it is one of the most used tags for recombinant proteins in bacterial cells [10,22].

Both OMP2c.cVLP and MBP.INVASc induced robust systemic antibody responses in mice and pigs. However, the relevance of antibody-mediated protection against LI and the protection associated with natural LI is not fully established [22]. These findings may suggest that the natural infection and current whole pathogen vaccines fail to generate antibody responses of sufficient magnitude towards specific surface antigens, rather than indicating a general inadequacy of humoral responses in preventing LI-induced enteric disease. Therefore, further investigation into the immunological correlations of protection and the relative importance and contribution of systemic and mucosal responses in controlling the pathogen will be highly beneficial. In this study, pigs were vaccinated i.m. Therefore, while strong humoral levels were detected in the serum, this approach likely did not generate gut-specific mucosal responses, which may be an additional factor that can be optimized in further studies.

In clinical trials, mild-to-moderate clinical signs of LI infection, such as soft feces or watery diarrhea, were observed across all groups (Figure 4D). This suggests that despite the vaccines induced an immune response, they did not fully prevent the clinical manifestations of the infection. However, the signs observed were mild, and the animals remained active. Additionally, the fact that the animals remained active despite exhibiting mild symptoms implies that the vaccines may help maintain general health and activity levels, even in the presence of infection. Those observations might be explained by inherent variability in the host response; therefore, a larger sample size in further experiments would improve the robustness of our findings. Furthermore, we observed a similar average daily gain among the groups (Figure 4A), which was slightly lower than the Danish reference daily average gain of 463 g [23]. A reduction in weight gain occurs when young piglets are primarily exposed to LI [5], with clinically affected animals reducing daily gains proportional to the level of bacterial shedding [21]. Infected enterocytes accumulate LI at the apical end, causing heavily infected cells to swell and form protrusions, and as the disease progresses, LI can also be observed in macrophages located in the lamina propria [4]. This leads to a marked hyperplastic proliferation of immature crypt-epithelial cells. In this study, the protection provided, particularly that provided by MBP.INVASc, was evident from the reduction in lesion severity, as well as the low detection of LI (low IHC scores). Although our study did not determine whether the vaccines could prevent bacterial shedding, a significant reduction in pathology scores was observed among the vaccinated animals (Figure 2). Both subunit vaccines (MBP.INVASc nor OMP2c.cVLP) demonstrated a specific effect in minimizing inflammation (Figure 2E,F), suggesting that these vaccines offer protection against inflammation. These findings suggest that although our subunit vaccines do not completely prevent bacterial shedding, they do confer a degree of protection by reducing the pathological impact of the infection. This aligns with observations from another study on inactivated or killed vaccines, which were also not effective in preventing bacterial shedding following exposure to a field strain but were demonstrated to reduce inflammation degree [24].

In natural infections, both humoral and cell-mediated responses occur, potentially providing a better mucosal response against colonization. Other studies have found similar limitations, likely due to the stimulation of mainly humoral response [25,26]. Perhaps further investigation of alternative routes, such as intranasal and oral dosing, could help overcome some limitations and mimic a natural infection. Unfortunately, similar studies using VLP vaccines have not succeeded with intranasal dosing in pigs [11]. Therefore, further development of vaccine alternative delivery methods is still required and strongly suggested.

Interestingly, our observations show slightly higher levels of haptoglobin among MBP.INVASc-vaccinated piglets, even though it is not significantly different (Figure 2C). Haptoglobin is an acute-phase protein that binds to free hemoglobin, thereby preventing oxidative damage [20]. The kinetics of its induction following bacterial infection are related to an acute-phase response, which is an indicator of early defense mechanisms unspecified against trauma, inflammation, or infection [19].

## 5. Conclusions

Our subunit OMP2c.cVLP and MBP.INVASc vaccines demonstrated the ability to induce antigen-specific immune responses in piglets. Although none of the vaccines convincingly prevented LI infection, they effectively reduced inflammation and pathogen presence in vaccinated piglets, especially the MBP.INVASc vaccine. Our findings suggest that both vaccines have the potential for further development and optimization to enhance their protective efficacy against LI infections.

## Data Availability

The datasets used or analyzed in this study are available from the corresponding authors upon reasonable request.

## Supplementary Materials

The following supporting information can be downloaded at: https://www.mdpi.com/article/10.3390/vaccines13020207/s1, Supplementary Figure S1: Characterization and QC of OMP2c.cVLP and INVASc (A) SDS-PAGE analysis of OMP2c.cVLP preparation. M: molecular weight marker; *lane 1*: Catcher.OMP2c under reduced conditions (37 kDa); *lane 2*: Catcher.OMP2c under non-reduced conditions showing a disulfide-dependent dimer population (74 kDa); *lane 3*: unconjugated Tag.cVLP subunits (16 kDa); *lane 4*: OMP2c.cVLP following overnight incubation of Catcher.OMP2c and Tag.cVLP resulting in the formation of a coupling band (53 kDa); *lane 5*: final OMP2c.cVLP vaccine following the removal of excess Catcher.OMP2c by ultracentrifugation. (B) Dynamic light scattering (DLS) analysis of OMP2c.cVLP. The average hydrodynamic diameter and percentage polydispersity (%Pd) is indicated. (C) Representative negative stain transmission electron microscopy (TEM) images of OMP2c.cVLP. Scale bar indicates 100 nm. (D). Reduced SDS-PAGE of the recombinant MBP.INVASc.
