# Supplementary material for: Outer Membrane Proteins as Vaccine Targets Against Lawsonia intracellularis in Piglets
Source: Vaccines (Basel). 2025 Feb 19;13(2):207. doi: 10.3390/vaccines13020207 (PMC11861839; doi:10.3390/vaccines13020207)
Supplement: Supplementary file 1 [file vaccines-13-00207-s001.zip › vaccines-3383678-supplementary.pdf]

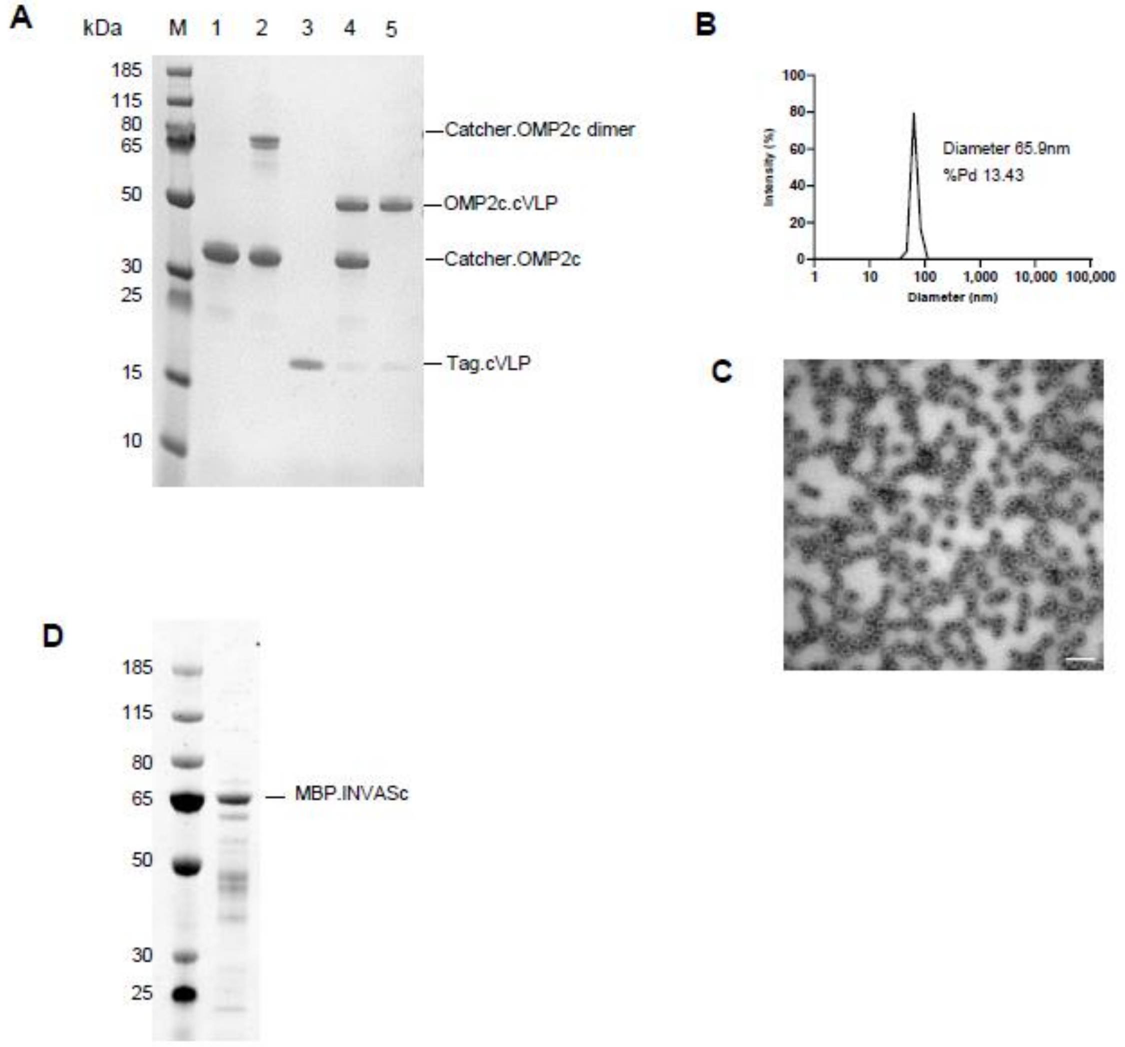

Supplementary Figure S1: Characterization and QC of OMP2c.cVLP and INVASc (A) SDS-PAGE analysis of OMP2c.cVLP preparation. M: molecular weight marker; *lane 1*: Catcher.OMP2c under reduced conditions (37 kDa); *lane 2*: Catcher.OMP2c under non-reduced conditions showing a disulfide-dependent dimer population (74 kDa); *lane 3*: unconjugated Tag.cVLP subunits (16 kDa); *lane 4*: OMP2c.cVLP following overnight incubation of Catcher.OMP2c and Tag.cVLP resulting in the formation of a coupling band (53 kDa); *lane 5*: final OMP2c.cVLP vaccine following the removal of excess Catcher.OMP2c by ultracentrifugation. **(B)** Dynamic light scattering (DLS) analysis of OMP2c.cVLP. The average hydrodynamic diameter and percentage polydispersity (%Pd) is indicated. **(C)** Representative negative stain transmission electron microscopy (TEM) images of OMP2c.cVLP. Scale bar indicates 100 nm. (**D**). Reduced SDS-PAGE of the recombinant MBP.INVASc.
